# Impact of Fear of Contracting COVID-19 and Complying with the Rules of Isolation on Nutritional Behaviors of Polish Adults

**DOI:** 10.3390/ijerph18041631

**Published:** 2021-02-09

**Authors:** Iwona Kowalczuk, Jerzy Gębski

**Affiliations:** Institute of Human Nutrition Sciences, Warsaw University of Life Sciences (SGGW-WULS), Nowoursynowska 159C, 02-776 Warsaw, Poland; iwona_kowalczuk@sggw.edu.pl

**Keywords:** COVID-19, nutritional behaviors, food purchases, eating patterns, changes in food product consumption, Poland

## Abstract

The aim of the study was to examine whether, and to what extent, fear of contracting Covid-19 and compliance with the mandatory rules of isolation affected Polish adults’ nutritional behaviors. The online study was carried out during the first wave of the pandemic on a sample of 926 adults. Through cluster analysis, three groups of respondents were isolated: 1. People who fear a Covid-19 infection and follow the isolation rules (FFR), 2. People moderately afraid of the disease and following the rules loosely (MFFR), 3. People who are not afraid of the infection and do not follow the rules of isolation. (NFFR). The clusters were profiled with consideration of different aspects of eating behaviors as well as socio-demographic and economic features. The results of the study show a close relationship between the level of fear of contracting Covid-19 and the degree to which isolation rules are followed. These two factors were found to have a significant impact on eating behaviors, such as food purchases, eating patterns, and levels of consumption. It was stated that the FFR group changed their eating behaviors the most in terms of food purchasing, eating habits (excluding diversity and quality of diet), and food product consumption. The greatest stability in the majority of the analyzed areas of nutritional behaviors was observed in the MFFR cluster. The NFFR group shown the greatest decrease both in regularity and quality of their meals. This group also exhibited a significant increase in the consumption of alcoholic beverages. The results of the study can be useful in the decision making process when introducing restrictions or managing information. They also point to the need for extensive nutritional education focused on explaining the relationship between nutrition and health during a pandemic.

## 1. Introduction

In the last twenty years, the world has faced many pandemics (SARS in 2002, H1N1 in 2009–2010, Ebola in 2014, and MERS in 2015), however, in comparison to these, the COVID-19 pandemic, which started in early 2020, appears to be particularly dangerous, due to its scale and rapid spread.

With the outbreak of the coronavirus disease, national polls in many countries indicate sharp increases in fear and worries relating to the virus [1,2]. Reactions of governments in countries affected by the pandemic have varied, however, a decisive majority have introduced mandatory social distancing, including lockdowns during peak pandemic periods [3].

In Poland, the first case of coronavirus was confirmed on 4 March 2020. Starting from 8 March, the first restrictions had been introduced (no mass events, closed borders, closed educational facilities), and the outbreak was announced as an epidemic on 20 March. The most restrictive social distancing measures were in force from 1–19 April, with restrictions being gradually lifted henceforth. The restrictions were introduced coupled with a fear of the Covid-19 disease, as well as a lack of verified information regarding the disease and its consequences, which caused significant distress among the people of Poland. A study conducted in May 2020 has shown that 69% of Poles are ‘very’ (26%) or ‘a little’ (43%) scared of contracting the coronavirus, whereas only 28% had declared that they are ‘rather’ (19%) or ‘not at all’ (9%) afraid of it [4].

Stress caused by both the imposed restrictions and the fear of contracting the disease can significantly alter people’s lifestyles [5], including eating behaviors. It should be emphasized that in situations where contracting a disease becomes a serious concern, proper nutrition becomes particularly important due to its importance for the body’s immunity [6,7,8,9].

Stress may affect the amount of food consumed as well as the attitude towards eating and the quality of the diet. As early as the 1990s, Greeno and Wing [10] noticed that, depending on the individual characteristics and predisposition, stress may cause either an increase or a decrease in food consumption. This observation has been confirmed in other studies [11,12,13]. It was also noted that people affected by stress feel less desire or motivation to eat and do not enjoy eating [14]. Increases in abnormal dietary behaviors under stressful circumstances have also been found [15]. Many studies [13,16,17,18] have shown that increased stress levels are linked not only to unhealthy eating patterns but also to the poor quality of the food consumed.

Previous studies also indicate the effect of forced isolation on eating behavior. Lockdown has a profound impact on food access [3], choice of places for grocery shopping and planning purchases [19,20], and spending on food products [21,22]. Being confined to one’s home directly affects their lifestyle, extending to patterns of physical activity, structure, and the amount of food consumed [23]. People in isolation are prone to developing irregular eating patterns and snacking frequently, which in turn can lead to a higher caloric intake and increase the risk of obesity [24].

Much research was undertaken in 2020 on eating behavior during the COVID-19 pandemic, and all of them show changes, although their results are sometimes conflicting. Celik and Dane [25] noticed that as a result of the pandemic, food preference has changed from “cost and health” to “quality and health”, and consumers’ interest in fruit and vegetable consumption has increased. Similarly, in a study conducted in China Luo et al. noticed that during this public emergency, almost 80% of participants changed their dietary habits, including increasing vegetables, fruit, and water intake, and reducing sugary drinks and snacks [26]. According to the results of research conducted by Pišot et al. [27] in nine European countries, during restriction measures as a result of the Covid-19 pandemic, more regular meals, larger meal sizes, less unhealthy food, and lower alcohol consumption was noted. Zupo et al. [28], after analyzing the results of 12 studies, noticed a rise in carbohydrate source consumption, especially those with a high glycemic index, as well as more frequent snacks. Higher consumption of fruits and vegetables, and protein sources, particularly pulses, was also recorded. Data concerning the consumption of junk foods lacked consistency, while there was a decrease in alcohol intake and fresh fish and seafood consumption.

There are many factors that can influence nutritional behavior during the Covid-19 pandemic, however, two of them, namely fear of getting sick and mandatory isolation, seem to be the most important. The aim of this study was to examine if there is a connection between fear of contracting Covid-19 and adherence to isolation rules, and whether, and to what extent, these two factors affected Polish adults’ nutritional behaviors.

Based on the literature information, as well as authors’ observations and experiences, five research hypotheses were formulated:

1. There is a relationship between the level of fear of contracting Covid-19 and the degree to which isolation rules are being observed.
2. The group with the highest fear level and isolation adherence consists predominantly of women, as well as young and elderly people.
3. The changes in eating behavior concern such areas as food purchases, eating patterns, and type of food product consumed.
4. The greatest changes in all of the analyzed areas of eating behavior occur in the group exhibiting the highest fear level and isolation adherence.
5. The most positive changes, from the health point of view, occur in the group exhibiting the highest fear level and isolation adherence.

## 2. Materials and Methods

The research was carried out from 20 March to 30 May 2020 on a sample of 926 adults (the characteristics of the study sample are displayed in Table 1).

### 2.1. Data Collecting

For collecting data, the snowball method was used. Based on the personal contacts of the authors, the initial group of respondents, consisting of 50 adult people (not suffering from COVID-19 or having been quarantined), was created. The group was analogous to the structure of the Polish population in terms of gender (women 52%, men 48%) and age (18–25 years old 12%, 26–35 years old 20%, 36–45 years old 18%, 46–55 years old 14%, 56–65 years old 18%, and 65 years old 18%). The respondents from the initial group also participated in the pilot test and validation of the research questionnaire. The validation process was carried out with the use of the Cronbach alpha reliability test (the α Cronbach’s coefficient was 0.82). The initial group members were asked to share the study link to persons who were, according to the sender’s knowledge, not suffering from COVID-19 or were not quarantined. The link was shared through social media, such as Facebook, and WhatsApp, and by personal contacts of the initial group members.

### 2.2. Research Questionnaire

The research questionnaire was created using the Google Forms web survey platform. A brief description of the study and its aim and the declaration of anonymity and confidentiality were given to the participants before the start of the questionnaire. Respondents did not provide their names or contact information (including the IP address) and could finish the survey at any stage. The answers were saved only by clicking the “submit” button after filling out the questionnaire.

The questionnaire consisted of three sections. The first one was based on the collection of socio-demographic data of the respondents including characteristics such as gender, age, education, place of residence, income, and professional activity during the COVID-19 pandemic.

The second part of the questionnaire contained two questions—the question about the fear of getting infected (on a 3 point scale: 1—I am very afraid of getting sick, 2—I am afraid of getting sick, 3—I am not afraid of getting sick) and the question about compliance with the restriction introduced (on a 3 point scale: 1—I definitely follow the isolation rules, 2—I rather follow the isolation rules, 3—I do not follow the isolation rules).

The third part of the questionnaire contained three questions regarding changes in nutritional behaviors of consumers during a pandemic. They concerned such aspects as: changes in the organization of food purchases (6 items in positional scale from 1—much less often to 5—much more often), changes in eating patterns (5 items on a positional scale from 1—much less to 5—much more), and changes in the level of consumption of 16 groups of food products, alcoholic beverages and supplements (on the positional scale from 1—I consume much less to 5—I consume much more).

### 2.3. Statistical Analysis

Spearman’s correlation was used to determine the strength of the association between the level of fear of contracting Covid-19 and the degree of adherence to isolation.

The cluster analysis was carried out based on the means of the answers to questions about the fear of getting infected and compliance with the restriction introduced. The hierarchical method was used to isolate the clusters. The number of clusters was chosen based on the dendrogram and pseudo-F and pseudo-T2 statistics. Chi-square independence test was used to evaluate significant differences among identified clusters.

The information obtained as a result of the answers to the questions from parts 1 and 3 of the questionnaire was used for profiling the respondent clusters. Chi-square analysis was used to determine significant differences between categorical variables (*p* < 0.05). All calculations were made using the SAS 9.4 statistics package (SAS Institute, Cary, NC, USA).

**Figure 1:**
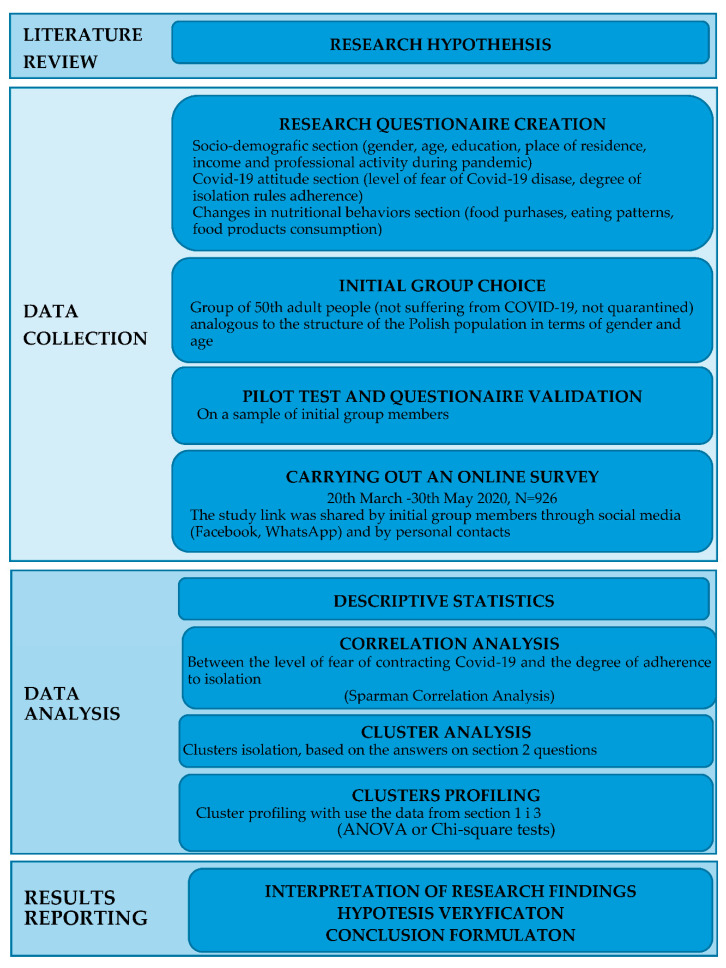
Research conception and methodology.

A flowchart describing the research conception and methodology is presented in Figure 1.

## 3. Results

Correlation analysis showed a strong connection (R = 0.58481, *p* < 0.0001) between the level of anxiety of contracting Covid-19 and the degree of adherence to isolation.

As a result of the cluster analysis, three accurately separated clusters were obtained:

1. Fearing and following the rules (FFR, N = 352, 38%)—persons who are very afraid of getting sick and follow the safety rules most strictly.
2. Moderately fearing and following the rules (MFFR, N = 400, 43.2%)—persons who are moderately afraid of getting sick and rather follow the safety rules.
3. Not fearing and not following the rules (NFFR, N = 174, 18.8%)—persons who are least afraid of getting sick and rather or do not follow the rules (Table 1).

### 3.1. Socio-Demographic and Economic Characteristics of the Study Population and Isolated Clusters

The results of the descriptive statistical analysis indicated that in the sample of 926 respondents, the majority were female (75.1%). In terms of age, the most numerous group consisted of respondents aged 26–35 years old (23.3%), while older groups of respondents were the least numerous (56–65 years old—12.7%, over 65 years old—7.7%). Other groups were represented by approximately 19% of the respondents. Most consumers had a high school diploma (66.9%). Nearly half of the respondents (48.5%) lived in large cities, and 20% in rural areas. Slightly more than half of the respondents (51.2%) earn up to PLN 3 000 (670 Euro), while the rest reported a higher income. The most numerous group of respondents during pandemic worked or studied remotely (35.2%), 20.4% declared that they partially work or study remotely, 19.6% worked or studied as before, 16.4% did not work or studied as earlier, and 8.4% did not work or studied as a result of the pandemic outbreak.

Socio-demographic analysis of the identified clusters showed that the FFR group, compared to the general population, included a higher percentage of women, people aged 36–45 and 46–55 years, and those with a University degree. This group also had a higher percentage of large city residents, reporting an income of PLN 3001–4000 and above, as well as people who did not work or study before the pandemic. The MFFR group had a relatively higher percentage of people aged 26–35 years, who lived in small towns, earned PLN 2001–3000 and less, as well as respondents who declared working or studying remotely or partially remotely. The NFFR group, in turn, was found to include a higher percentage of men than the general population, as well as people from the youngest age groups and those with primary and secondary education. The NFFR group also had more residents of rural areas, earning PLN 2001–3000, and people who declared that they worked or studied as before, or did not work/study as a result of the pandemic. In terms of age, this group had a higher percentage of young people aged 26–35 years, but also a larger share of the oldest respondents (Table 2).

### 3.2. Changes in Food Purchases

**Figure 2:**
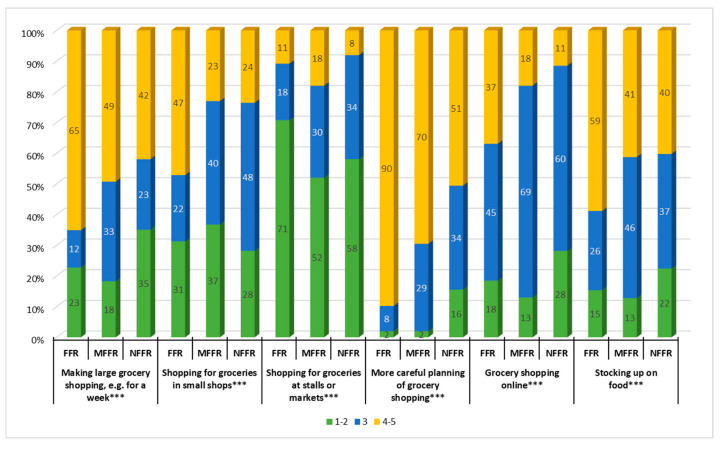
Changes in food purchases. FFR—Fearing and following the rules, MFFR—Moderately fearing and following the rules, NFFR—Not fearing and not following the rules. The direction of changes: 1—much less often, 2—less often, 3—no changes, 4—more often, 5—much more often. Statistically significant in the chi-square test at: *** < 0.0001

Across all study groups, an increase in the tendency to plan shopping, make large purchases, and stock up on food, a slight increase in online purchases, and a decline in interest to buy at stalls and markets were found. Statistically significant differences were discovered between the identified clusters concerning all analyzed aspects of shopping behavior. Representatives of the FFR cluster made large purchases, bought food in small shops and online, planned their grocery shopping and stocked up on food more often than other respondents, and were less likely to buy at stalls or markets. The MFFR cluster exhibited the least profound changes in their shopping habits. Compared to other clusters, they were slightly less likely to purchase food in small shops but bought at markets more often. In turn, the NFFR group were less likely to make large purchases and shop for groceries online. They also showed a relatively low increase in the careful planning of food purchases (Figure 2).

### 3.3. Changes in Eating Patterns

**Figure 3:**
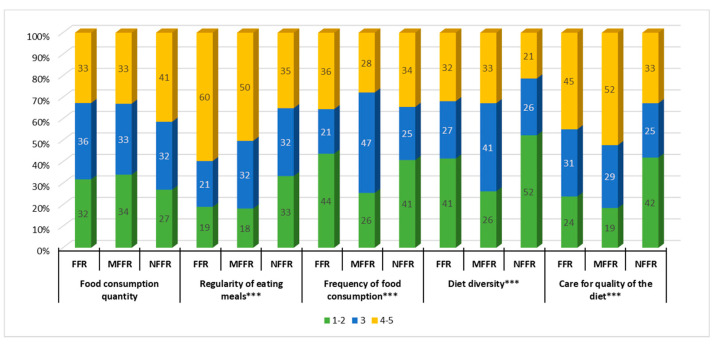
Changes in eating patterns. FFR—Fearing and following the rules, MFFR—Moderately fearing and following the rules, NFFR—Not fearing and not following the rules. The direction of changes: 1—much less, 2—less, 3—no changes, 4—more, 5—much more, Statistically significant in the chi-square test at: *** < 0.0001.

The analysis of changes in eating habits in the general study population showed that almost one-third of the respondents reported a decrease in the quantity of food consumed, and a similar percentage declared an increase. No statistically significant differences in this matter between the identified clusters were found. More than half of the respondents reported eating more regularly, including almost 60% of the FFR cluster and only 35% of the NFFR cluster, who in turn reported a less regular eating pattern more often than other participants. Frequency in food consumption changed to the greatest extent for the FFR group, i.e.,35.5% of them declared an increase and 43.8% a decrease in eating frequency. Relatively large changes in this aspect were also found in the NFFR group, while the MFFR cluster showed the least change. A decrease in diversity of the diet was reported most often by respondents from the NFFR cluster, whereas representatives of FFR and MFFR clusters were more likely to declare an increase in this area. Quality of nutrition started to play a less significant role to the NFFR cluster, the MFFR group declared paying more attention to this matter since the beginning of the pandemic. The FFR cluster showed the least change in this area (Figure 3).

### 3.4. Changes in the Level of Food Product Consumption

The most significant changes in consumption levels across all study groups (less than 30% of ‘3—no changes’ marks) were recorded in cases of products such as alcoholic beverages, sweets, animal fats, vegetables, and dietary supplements. The largest increase in consumption (over 50% of ‘4’ and ‘5’ marks) was recorded for sweets, animal fats, alcoholic beverages, and dietary supplements, whereas a significant increase (over 30% of ‘4’ and ‘5’ marks) was found in case of fruits, vegetables, eggs, mineral water, and cereal products. On the other hand, the biggest decrease in consumption (over 50% of ‘1’ and ‘2’ marks) affected snacks, while significant decreases (over 30% of ‘1’ and ‘2’ marks) were recorded for vegetables, soft drinks, and alcoholic beverages. Between the identified clusters, a statistically significant variation of changes in consumption was recorded for all of the analyzed food products, except for vegetables.

**Figure 4:**
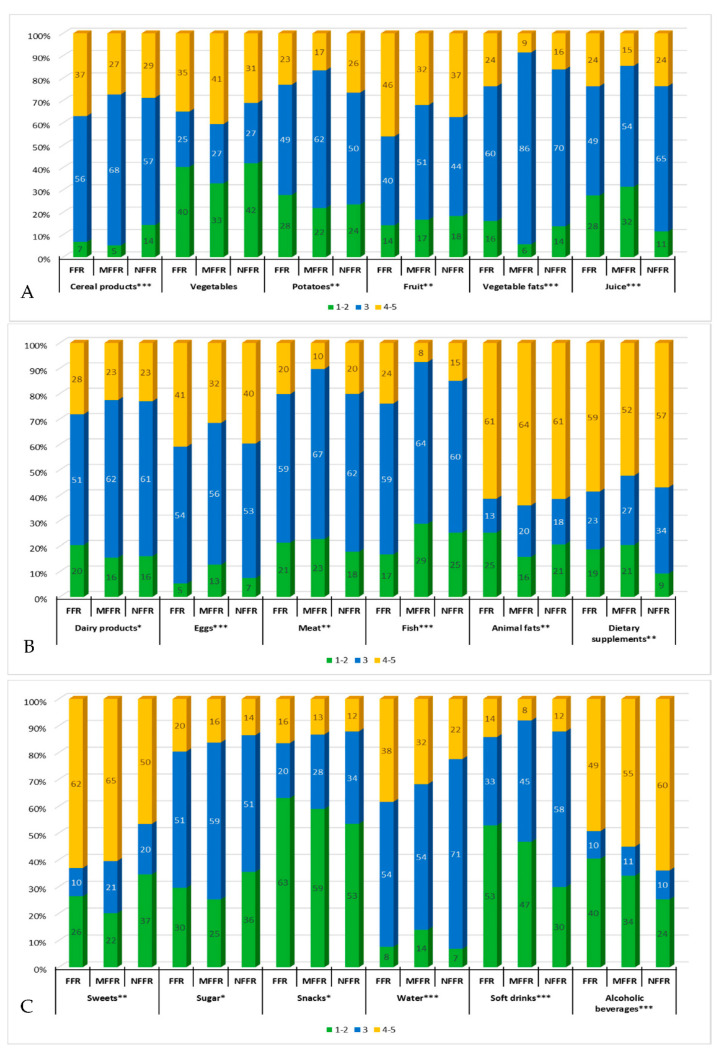
(**A**–**C**). Changes in the level of food product consumption, alcoholic beverages, and dietary supplements. FFR—Fearing and following the rules, MFFR—Moderately fearing and following the rules, NFFR—Not fearing and not following the rules. The direction of changes: 1—much less, 2— less, 3—no changes, 4—more, 5—much more. Statistically significant in the chi square test at: *** < 0.0001, ** < 0.01, * < 0.05.

The highest intensity of changes in consumption (lowest percentage of ‘3’ marks) was found in the FFR cluster. Moreover, this group declared an increase in consumption of all the analyzed food products more often than other clusters, except for alcoholic beverages, animal fats, meat, juices, vegetables, and potatoes. They also showed the largest decrease in the consumption of animal fats, alcoholic beverages, snacks, soft drinks, dairy products, potatoes, and vegetable oils. The MFFR cluster exhibited the highest stability of consumption for most of the products, except for beverages (mineral water, soft drinks, juice, alcohol) and dietary supplements. This group also had the highest percentage of declared decrease in eggs, meat, fish, juice, and dietary supplement consumption, and an increase in sweets and animal fats. The NFFR cluster exhibited a relatively higher stability of beverage (mineral water, soft drinks, juice) and dietary supplement consumption. Compared to other clusters, the NFFR was found to include a larger percentage of people who had limited their consumption of cereal products, fruits, sweets, and sugar, as well as a higher percentage of respondents that declared increased consumption of alcoholic beverages (Figure 4A–C).

## 4. Discussion

The study was an attempt to find out if, and to what extent, fear of contracting COVID-19 and abiding by the restrictions introduced affected eating behaviors of Polish adults during the first wave of the Covid-19 pandemic.

A close association was found between fear of contracting Covid-19 and compliance with the isolation rules, providing positive verification of hypothesis 1.

Through cluster analysis, three consumer groups were identified: fearing and following the rules (FFR), 38% of the general studied population; moderately fearing and following the rules (MFFR), 43.2%; not fearing and not following the rules (NFFR), 18.8%.

The identified clusters were diverse in terms of gender, age, place of residence, education, income, and professional activity during the pandemic. The FFR cluster was found to include a higher percentage of women, middle-aged people, respondents with a university degree, large city residents, people with higher income, as well as respondents who did not work or study either before or during the pandemic. Among the MFFR, there were relatively more people aged 56–65 years, small-town residents with lower income, as well as respondents who declared working/studying remotely or partially remotely. The NFFR cluster had a higher percentage of men and both the youngest and oldest study participants, as well as respondents with primary and secondary education, rural area residents, people with medium income, and those declaring that their work or studies were unaffected by the pandemic, or stopped by it altogether. The results of the study only partially confirmed the assumptions contained in hypothesis 2, regarding a greater share of women, as well as young and older people in the group with the highest fear level and isolation compliance.

Obtained results confirm the fact, also noted in other studies, that women exhibit a higher level of anxiety both during the pandemic [27] and in other stressful life events [28,29], as well as are more inclined to care about their health [30]. The conducted study also confirms an observation from prior research [31], that education level and place of residence affect attitudes toward the pandemic. Contrary to expectations, the oldest age group and male respondents were less likely to experience anxiety, even though these factors are linked to a higher mortality rate [32]. The results differ from the outcome of a previous study [31], according to which pandemic-related stress affects young people the most.

Analysis of the impact of attitudes towards the COVID-19 pandemic on eating behaviors explored such aspects as: food purchases, eating patterns, and the level of consumption for specific food products. Statistically significant differences between the isolated clusters were recorded in all of these areas, which confirms hypothesis 3.

The FFR cluster was found to make large grocery shopping more frequently, buy in small shops and online more often and less frequently at markets. They planned their shopping more carefully and were more likely to stock up on food. This group also exhibited the largest increase in eating regularity, as well as the biggest changes (both increase and decrease) in terms of frequency of eating meals and diversity thereof. The FFR cluster also declared the greatest intensity of changes in the level of consumption of all analyzed food products, including both the largest increase in their consumption (except for alcoholic beverages, animal fats, meat, juices, vegetables, and potatoes) and the biggest decrease in consumption of such products as dairy, vegetables, and animal fats, snacks, alcoholic beverages, and soft drinks.

The MFFR group reported the smallest changes in grocery shopping organization, a relatively high increase in care for the quality of nutrition, and a greater diversity of meals. This cluster was also found to exhibit the greatest stability of consumption for most food products, except for beverages (mineral water, soft drinks, juice, alcoholic beverages) and dietary supplements. Members of this group limited largely their consumption of eggs, meat and fish, juice, and dietary supplements, but increased their sweets and animal fats intake.

People in the NFFR cluster were less likely to plan their grocery shopping and make large purchases or shop online less often. This group more often declared a decrease in regularity of their meals, as well as a lesser diversity of their diet and paying less attention to the quality of nutrition. They showed relatively higher stability of beverage and dietary supplements consumption, a significant increase in consumption of alcoholic beverages, and a decline in consumption of cereal products, fruit, sweets, and sugar.

These differences in changes in nutritional behavior between the clusters confirm hypothesis 4, namely that the greatest changes in eating behavior occur in the group of those with the highest fear level and compliance with isolation rules.

Due to the particular approach this study takes toward the issue, the obtained results can be only partially compared to the results of previous research on changes in nutritional behavior during the Covid-19 pandemic. Nevertheless, the results do confirm an increased likelihood of making large purchases, planning shopping more carefully, and stocking up on food, found also by Jribi et al. [19], as well as an increased interest in online shopping noticed previously by Jung et al. [20].

Results of the study show that people most afraid of the disease and most compliant with the isolation rules (and presumably experiencing the highest stress levels) exhibit the highest polarization of changes in nutritional behaviors. This kind of ‘bipolar’ reaction to stress has also been observed in other studies [11,12,13].

Many studies [13,15,16,17,18] highlight the link between stress and intensification of behaviors, contributing to a bad diet. This study only partially confirmed this connection, even though the greatest increase in consumption of sweets, sugar, snacks, and soft drinks was noted in the FFR group, but its members also declared the largest decrease in the intake of some of the previously mentioned products (snacks, soft drinks), as well as in consumption of animal fats and alcoholic beverages.

Existing studies on changes in nutritional behavior during the Covid-19 pandemic [23,24,25] proved that the pandemic has triggered a more health-conscious approach to nutrition. This study confirmed this observation only partially. Admittedly, an increased consumption was observed for products beneficial to health (e.g., vegetables, fruits, mineral water) but it was coupled with a higher intake of sweets, sugar, animal fats, alcoholic beverages, and diet supplements, which, according to many studies [33,34,35,36,37], may have a negative impact on health.

As can be seen from the above considerations, the obtained results do not allow for unambiguous verification of hypothesis 5.

## 5. Limitations and Further Research

Two main limitations of the conducted study can be noticed. The first one concerns the methodology. The study was carried out using the online survey method, because of specific conditions during the pandemic, as remote data collection using social networks is feasible and necessary in such cases [38].

The respondents were recruited using the snowball method, which is not a random one. However, the authors wanted to reach out to people who were not suffering from COVID-19 or were not quarantined, and it would be an ethical abuse to mention this in the survey questionnaire. It was also assumed that social interactions between study participants would oblige them to provide truthful answers. Unfortunately, the way of the sample selection does not allow for full control of its structure, and the methods of the questionnaire sharing eliminate people with limited Internet skills or Internet access from the response base. For these reasons, respondents were predominantly women, young persons, and people with higher education. Because the study sample was not representative of the Polish adult population, the results of this study cannot be generalized.

The second limitation concerns the way of determining changes in eating behavior, i.e., this study did not attempt to determine the absolute value of declared changes, and only their direction, which can be considered a general and preliminary recognition of the problem.

In the authors’ opinion, the matter of the impact of the Covid-19 pandemic on nutritional behaviors ought to be subjected to further studies, which should focus on a more precise determination of the scale of changes and their possible consequences to health. It would also be worthwhile to compare the reactions of Poles with those of other nations and determine how long the changes in eating behavior observed in the study will last.

## 6. Conclusions

The results of the study show a close relationship between the level of fear of contracting Covid-19 and the degree to which isolation rules are followed. A significant influence of both these factors on eating behavior was also noticed.

It was stated that the group of respondents most afraid of contracting the disease and who followed the isolation rules most strictly, changed their eating behaviors the most c both in terms of food purchasing, eating habits (excluding diversity and quality of diet), and food product consumption. The greatest stability in the majority of the analyzed areas of nutritional behaviors was observed in the group moderately afraid of Covid-19 infection and moderately abiding by the isolation rules. The group declaring the lowest level of fear and the lowest compliance with the isolation rules has shown the greatest decrease both in regularity and quality of their meals. This group also exhibited a significant increase in the consumption of alcoholic beverages.

These results can be useful in the decision making process when introducing restrictions or managing information. They also point to the need for extensive nutritional education focused on explaining the relationship between nutrition and health during a pandemic.

## Figures and Tables

**Table 1:** Study population and isolated cluster characteristics including fear of getting sick and compliance with isolation rules.

Variable	Level of Variable	Total	MFR *	FFR *	NFFR *	*p*-Value	Cramer V
	n/%	926/100	400/43.2	352/38	174/18.8		
Compliance with isolation rules	I definitely follow the rules	367	95/23.8	271/77.0	1/0.6	<0.0001	0.80
	I rather follow the rules	385	303/75.7	80/22.7	2/1.1		
	I rather or do not follow the rules	174	2/0.5	1/0.3	171/98.3		
Fear of getting sick	I am very afraid of getting sick	373	2/0.5	350/99.4	21/12.1	<0.0001	0.77
	I am a bit afraid of getting sick	431	365/91.2	1/0.3	65/37.4		
	I am not afraid of getting sick	122	33/8.3	1/0.3	88/50.5		

* FFR—Fearing and following the isolation rules, MFFR—Moderately fearing and following the isolation rules, NFFR—Not fearing and following the isolation rules. Chi-square test of independence, *p*-value < 0.05—differences between groups are significant.

**Table 2:** Study population and isolated cluster characteristics including socio-demographic and economic features.

Specification	Total	FFR *	MFFR	NFFR	*p*
Gender					
Woman	75.05	83.52	73.25	62.07	<0.0001
Man	24.95	16.48	26.75	37.93	
Age					
18–25 y.o.	18.79	15.63	21.00	20.11	<0.0001
26–35 y.o.	23.33	21.31	23.75	26.44	
36–45 y.o.	18.68	23.30	14.25	19.54	
46–55 y.o.	18.79	24.43	19.00	6.90	
56–65 y.o.	12.74	8.81	18.00	8.62	
over 65 y.o.	7.67	6.53	4.00	18.39	
Place of residence					
Rural area	20.73	19.89	17.50	29.89	<0.0001
Town up to 10,000 people	11.45	9.38	14.25	9.20	
Town between 10,000 and 500,000 people	19.33	16.76	20.25	22.41	
City over 500,000 people	48.49	53.98	48.00	38.51	
Income					
Up to PLN 2000 (448€) **	21.16	21.03	22.25	19.96	<0.0001
PLN 2001–3000 (449–673€)	30.02	22.44	35.50	34.06	
PLN 3001–4000 (674–897€)	24.30	32.39	19.75	22.99	
Over PLN 4000 (897€)	24.52	24.16	22.50	22.99	
Education					
Primary/vocational	3.89	2.84	2.50	9.20	<0.0001
Secondary	29.27	25.28	30.75	33.91	
Higher	66.85	71.88	66.75	56.90	
Professional activity during the COVID-19 pandemic					
I work/study as before (no changes)	19.55	12.50	19.25	34.48	<0.0001
I work/study remotely	35.21	36.65	40.25	20.69	
I partially work/study remotely	20.41	20.17	21.25	18.97	
I do not work/study during the pandemic	8.42	7.10	8.25	11.49	
I do not work/study as before (no changes)	16.41	23.58	11.00	14.37	

* FFR—Fearing and following the rules, MFFR—Moderately fearing and following the rules, NFFR—Not fearing and not following the rules. ** Euro exchange rate at the National Bank of Poland on 30 September 2020. Chi-square test of independence, *p*-value < 0.05—differences between groups are significant.

## Data Availability

Principles of data transparency have been respected. The survey questionnaire and coded survey data are available by request to corresponding author.
